# Genetically Proxied Inhibition of Coagulation Factors and Risk of Cardiovascular Disease: A Mendelian Randomization Study

**DOI:** 10.1161/JAHA.120.019644

**Published:** 2021-04-09

**Authors:** Shuai Yuan, Stephen Burgess, Mike Laffan, Amy M. Mason, Martin Dichgans, Dipender Gill, Susanna C. Larsson

**Affiliations:** ^1^ Unit of Cardiovascular and Nutritional Epidemiology Institute of Environmental Medicine Karolinska Institutet Stockholm Sweden; ^2^ Department of Public Health and Primary Care University of Cambridge Cambridge United Kingdom; ^3^ MRC Biostatistics Unit University of Cambridge United Kingdom; ^4^ Centre for Haematology Imperial College London United Kingdom; ^5^ British Heart Foundation Cardiovascular Epidemiology Unit Department of Public Health and Primary Care University of Cambridge United Kingdom; ^6^ National Institute for Health Research Cambridge Biomedical Research Centre University of Cambridge and Cambridge University Hospitals Cambridge United Kingdom; ^7^ Institute for Stroke and Dementia Research University Hospital LMU Munich Germany; ^8^ Munich Cluster for Systems Neurology (SyNergy) Munich Germany; ^9^ German Centre for Neurodegenerative Diseases (DZNE, Munich) Munich Germany; ^10^ Department of Biostatistics and Epidemiology School of Public Health Imperial College London United Kingdom; ^11^ Clinical Pharmacology and Therapeutics Section Institute of Medical and Biomedical Education and Institute for Infection and Immunity St George’s, University of London United Kingdom; ^12^ Clinical Pharmacology Group, Pharmacy and Medicines Directorate St George’s University Hospitals NHS Foundation Trust London United Kingdom; ^13^ Centre for Pharmacology & Therapeutics Department of Medicine Hammersmith Campus Imperial College London United Kingdom; ^14^ Novo Nordisk Research Centre Oxford Oxford United Kingdom; ^15^ Unit of Medical Epidemiology Department of Surgical Sciences Uppsala University Uppsala Sweden

**Keywords:** cardiovascular disease, coagulation, Mendelian randomization analysis, stroke, venous thromboembolism, Ischemic Stroke, Thrombosis

## Abstract

**Background:**

We conducted Mendelian randomization analyses investigating the linear associations of genetically proxied inhibition of different coagulation factors with risk of common cardiovascular diseases.

**Methods and Results:**

Genetic instruments proxying coagulation factor inhibition were identified from genome‐wide association studies for activated partial thromboplastin time and prothrombin time in BioBank Japan (up to 58 110 participants). Instruments were identified for 9 coagulation factors (fibrinogen alpha, beta, and gamma chain; and factors II, V, VII, X, XI, and XII). Age‐ and sex‐adjusted estimates for associations of the instruments with the outcomes were derived from UK Biobank and the FinnGen, CARDIoGRAMplusC4D (Coronary Artery Disease Genome‐wide Replication and Meta‐analysis), and MEGASTROKE consortia with numbers of incident and prevalent cases of 820 to 60 810. Genetically proxied inhibition of fibrinogen alpha, beta, and gamma chain, factor II, and factor XI were associated with reduced risk of venous thromboembolism (*P*<0.001). With the exception of fibrinogen beta and factor II, inhibition of these factors was also associated with reduced risk of any ischemic stroke and cardioembolic stroke (*P*≤0.002). Genetically proxied inhibition of fibrinogen beta and gamma were associated with reduced large‐artery stroke risk (*P*=0.001). There were suggestive protective associations of genetically proxied inhibition of factors V, VII, and X with ischemic stroke (*P*<0.05), and suggestive adverse associations of genetically proxied inhibition of factors II and XII with subarachnoid hemorrhage.

**Conclusions:**

This study supports targeting fibrinogen and factor XI for reducing venous thromboembolism and ischemic stroke risk, and showed suggestive evidence that inhibition of factors V, VII, and X might reduce ischemic stroke risk.

**Nonstandard Abbreviations and Acronyms**
aPTT: activated partial thromboplastin time
MR: Mendelian randomization

**Clinical Perspective**

**What Is New?**

- Genetically proxied inhibition of fibrinogen alpha, beta, and gamma chain and factors II and XI were associated with reduced risk of venous thromboembolism.
- With the exception of fibrinogen beta and factor II, inhibition of these factors was also associated with reduced risk of any ischemic stroke and cardioembolic stroke.
- Genetically proxied inhibition of fibrinogen alpha and gamma were associated with reduced large‐artery stroke risk.

**What Are the Clinical Implications?**

- The present Mendelian randomization study supports the efficacy of anticoagulants targeting fibrinogen, factor II, and factor XI in treating venous thromboembolism and revealed potential applications of inhibition of fibrinogen and factor XI for lowering risk of ischemic stroke, particularly cardioembolic stroke.
- Increased bleeding risk accompanied by these anticoagulants needs to be carefully assessed in further studies.

By inhibiting components of the coagulation cascade, current anticoagulant therapies have proven effective for the prevention and treatment of venous thromboembolism (VTE).^1^, ^2^ Anticoagulant therapies also reduce risk of other cardiovascular diseases (CVDs) via effects on thrombosis,^3^, ^4^, ^5^, ^6^ with the adverse consequence of increasing risk of bleeding complications.^4^ However, given large differences in the role of individual coagulation factors in CVD subtypes, the efficacy and safety of anticoagulants targeting different coagulation factors remains largely unknown.^7^ Recent meta‐analyses of randomized clinical trials aimed to compare the health benefits, adverse effects, and cost‐effectiveness across different anticoagulant drugs,^6^, ^8^ thereby informing on optimized anticoagulant treatment strategies. However, with the exception of approved antithrombotic agents such as vitamin K antagonists and factor IIa and factor Xa inhibitors, these questions could not be satisfactorily addressed because of insufficient availability of clinical trial data.^8^

By employing genetic variants as instrumental variables for coagulation factors in a Mendelian randomization (MR) framework, the clinical effects of inhibition of different pathways of the coagulation cascade can be assessed using observational data. This approach can strengthen the causal inference in an exposure‐outcome association by reducing residual confounding and reverse causality.^9^, ^10^ Because genetic variants are randomly allocated at conception, values of the exposure predicted by genetic variants are generally not correlated with other environmental factors, thus minimizing confounding. This process resembles the random assignment of participants to treatment and control groups in a randomized controlled trial. In addition, alleles of genetic variants are fixed at birth and cannot be modified by the onset or progression of the disease, and thus, the MR design also diminishes risk of reverse causality.

Previous and ongoing clinical trials have aimed to assess effects of different anticoagulants on atherosclerotic, thrombotic, and hemorrhagic CVDs. Anticoagulant drugs targeting a coagulation factor have been approved or are under investigation (Table 1). Clinical trials of oral anticoagulants often use intracranial hemorrhage, which includes intracerebral hemorrhage, subarachnoid hemorrhage, and epidural and subdural bleeds, as a measure for bleeding complications. Given that the latter 2 of these bleeding complications are rare, intracerebral and subarachnoid hemorrhage were used to evaluate bleeding complications in the present investigation.

**Table 1:** Coagulation Factors Included in This Mendelian Randomization Study and Information on Genetic Instrument Selection

Factor	Name	Related Measures	Gene	Chromosome	Position Start*	Position End*	SNPs	Previous MR Evidence	Approved Anticoagulants
I	Fibrinogen alpha chain	aPTT & PT	FGA	4	155504280	155511897	1	Yes	No
	Fibrinogen beta chain	aPTT & PT	FGB	4	155484132	155493915	1		No
	Fibrinogen gamma chain	aPTT & PT	FGG	4	155525286	155533902	1		No
II	Prothrombin	aPTT & PT	F2	11	46740743	46761056	1	No	Dabigatran, heparin, and enoxaparin
V	Proaccelerin or labile factor	aPTT & PT	F5	1	169481192	169555769	3	No	No
VII	Proconvertin or stable factor	PT	F7	13	113760102	113774995	1	Yes	No
X	Stuart‐Prower factor	aPTT & PT	F10	13	113777113	113803843	1	Yes	Warfarin, apixaban, enoxaparin, rivaroxaban, dalterparin, nadroparin calcium, fondaparinux sodium
XI	Plasma thromboplastin antecedent	aPTT	F11	4	187187099	187210835	1	Yes	No
XII	Hageman factor	aPTT	F12	5	176829139	176836577	1	No	No
…	aPPT	aPPT	…		…	…	19	No	…
…	PT	PT	…		…	…	15	No	…

aPTT indicates activated partial thromboplastin time; MR, Mendelian randomization; PT, prothrombin time; and SNP, single‐nucleotide polymorphism.

* Based on genome build GRCh37/hg19.

Here, we conducted a 2‐sample MR study to comprehensively assess the potential effect of inhibiting 9 coagulation factors on risk of 9 thrombosis‐related CVDs, including VTE, ischemic stroke and its etiologic subtypes, coronary artery disease, peripheral arterial disease, and intracerebral and subarachnoid hemorrhage. Secondary outcomes included heart failure, atrial fibrillation, aortic valve stenosis, and abdominal aortic aneurysm.

## Methods

### Study Design

**Figure 1:**
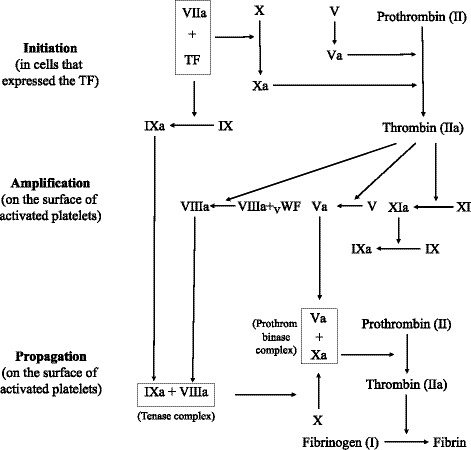
Coagulation factors involved in cell‐based coagulation cascade. Activated partial thromboplastin time is used to evaluate the coagulation factors Ⅻ, Ⅺ, Ⅸ, Ⅷ, Ⅹ, Ⅴ, Ⅱ, and Ⅰ. Prothrombin time evaluate the coagulation factors Ⅶ, Ⅹ, Ⅴ, Ⅱ, and Ⅰ. TF, tissue factor; VWF, von Willebrand factor.

For coagulation factors involved in cell‐based coagulation cascade (Figure 1),^11^ we identified genetic proxies as single‐nucleotide polymorphisms (SNPs) associated with activated partial thromboplastin time (aPTT) or prothrombin time (PT) and located within the gene region of the corresponding factor. We only used *cis*‐variants (variants in the relevant coding gene region) as genetic proxies. To explore whether the effects are target specific, we also selected SNPs associated with aPTT or PT and examined their associations with outcomes in supplementary analyses. Summary‐level data for aPTT and PT were obtained from BioBank Japan on 37 767 and 58 110 individuals, respectively.^12^ Genetic instruments were identified for the 9 coagulation factors, aPTT, and PT listed in Table 1 (there were no suitable SNPs for factor VIII and IX in their coding gene region). Outcome data were obtained from UK Biobank^13^ and the FinnGen,^14^ MEGASTROKE,^15^ and CARDIoGRAMplusC4D (Coronary Artery Disease Genome‐wide Replication and Meta‐analysis)^16^ consortia. All studies had obtained ethical approval, and participants had given informed consent.

### Genetic Instrument Construction

We constructed a genetic instrument consisting of SNPs associated with aPTT or PT at *P*<5×10^8^. For coagulation factors in the common pathway (ie, factors II, V, and X), the weights of genetic instruments from the association with PT were applied because no SNP associated with aPTT was identified at *P*<5×10^‐8^. Independent SNPs (*r^2^*<0.01 and clumping window >10 000) within the coding gene region of the corresponding coagulation factor were used as instrumental variables. Linkage disequilibrium *r^2^* and clumping window were estimated based on 1000 Genomes linkage disequilibrium reference panel (with only European population) and were obtained using the ld_matrix command in the TwoSampleMR package.^17^ SNPs in linkage disequilibrium within a particular window were pruned, and the SNP with the lowest *P* value was retained. The SNPs used as instrumental variables are shown in Table S1.

### Outcome Sources

The UK Biobank study^13^ was used to estimate genetic association with 10 CVDs, including the 6 major outcomes (VTE, ischemic stroke, coronary artery disease, peripheral arterial disease, and intracerebral and subarachnoid hemorrhage), among 367 643 adults (37–73 years of age at baseline) of European ancestry after exclusion of relatedness of third degree or higher, low genotype call rate (≥3 SDs from the mean), and excess heterozygosity. The participants were followed until March 31, 2017, or the date of death (recorded until February 14, 2018) with a median follow‐up of 8.0 years. CVD diagnosis was based on electronic health records, hospital procedure codes, and self‐reported information validated by interview with a nurse (Table S2). Clinical outcomes were not adjudicated by independent committee by predefined criteria. Beta coefficients and standard errors of the genetic associations with CVD were calculated using logistic regression with adjustment for age, sex, and 10 genetic principal components.

Publicly available summary‐level data were obtained for VTE and hemorrhagic stroke from the FinnGen consortium,^14^ for ischemic stroke from the MEGASTROKE consortium,^15^ and for coronary artery disease from the CARDIoGRAMplusC4D consortium.^16^ The FinnGen consortium R4 includes 6913 VTE cases, 1224 intracerebral hemorrhage cases, 1019 subarachnoid hemorrhage cases, and >163 500 noncases of Finnish descent. Association tests were adjusted for age, sex, 10 genetic principal components, and genotyping batch. The MEGASTROKE consortium includes 34 271 ischemic stroke cases and 404 630 noncases of European ancestry, and 4373 large‐artery stroke cases, 5386 small‐vessel stroke cases, and 7193 cardioembolic stroke cases. The MEGASTROKE consortium had some participant overlap with the FinnGen consortium, and therefore FinnGen was not used for analyses of ischemic stroke. The CARDIoGRAMplusC4D consortium involves 60 801 individuals with coronary heart disease and 123 504 noncases (77% of participants were of European ancestry). All SNPs used as instrumental variables for the coagulation factors were available in all outcome data sources. Diagnostic information for outcomes in FinnGen and consortia is presented in Tables S3 and S4.

### Statistical Analysis

The fixed‐effects inverse‐variance weighted method was used to assess the associations of coagulation factors with 10 CVDs (5 primary and 5 secondary outcomes) in the main analysis.^18^ Estimates were combined from different data sources using the fixed‐effects meta‐analysis method. The invariance weighted method with random effects was used to estimate the association of genetically predicted aPTT and PT with CVDs. All odds ratios and 95% CIs of the CVD outcomes were scaled to a 1‐second increase in aPTT and PT. The Bonferroni correction method was used to account for multiple testing. We deemed associations with *P*<0.001 (where *P*=0.05/54 [6 primary outcomes and 9 coagulation factors]) as strong evidence of causal associations. Associations with *P*<0.05 but >0.001 were treated as suggestive evidence of associations. Analyses were performed using the mrrobust package^19^ in Stata/SE 15.0 (StataCorp, College Station, TX) and the MendelianRandomization^20^ and TwoSampleMR^17^ packages in R software 3.6.0 (R Foundation for Statistical Computing, Vienna, Austria).

### Pleiotropy Assessment

To detect possible pleiotropic effects of used SNPs for coagulation factors, we searched used genetic instruments in PhenoScanner V2 (a database of human genotype‐phenotype associations)^21^ to obtain associated phenotypes at the genome‐wide significance level. Pleiotropy could not be explored statistically using MR sensitivity analyses because of a limited number of SNPs for each coagulation factor.

### Data Availability

The data that support the findings of this study are available from the corresponding author upon reasonable request.

## Results

**Figure 2:**
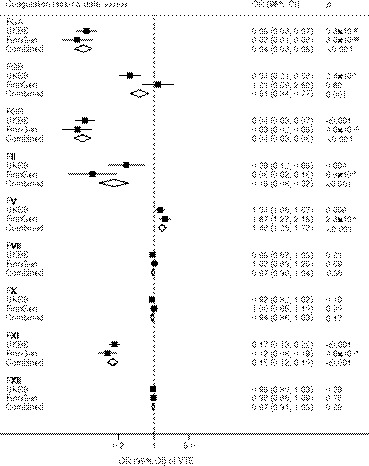
Genetically proxied inhibition of coagulation factors and venous thromboembolism (15 602 cases in UK Biobank and 6913 cases in FinnGen) in the main analysis. FGA indicates fibrinogen alpha chain; FGB, fibrinogen beta chain; FGG, fibrinogen gamma chain; IS, ischemic stroke; OR, odds ratio; UKBB, UK Biobank; and VTE, venous thromboembolism. Combined estimates were estimated using fixed‐effect meta‐analysis method.

**Figure 3:**
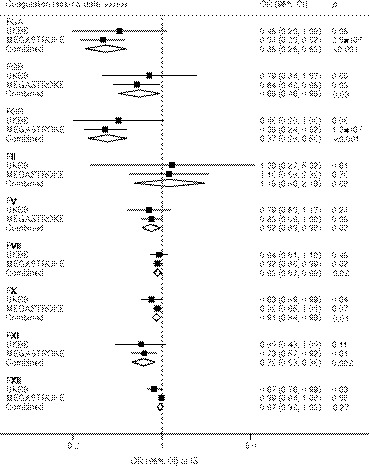
Genetically proxied inhibition of coagulation factors and ischemic stroke (4602 cases in UKBB and 34 272 cases in MEGASTROKE) in the main analysis. FGA indicates fibrinogen alpha chain; FGB, fibrinogen beta chain; FGG, fibrinogen gamma chain; IS, ischemic stroke; OR, odds ratio; and UKBB, UK Biobank. Combined estimates were estimated using fixed‐effect meta‐analysis method.

**Figure 4:**
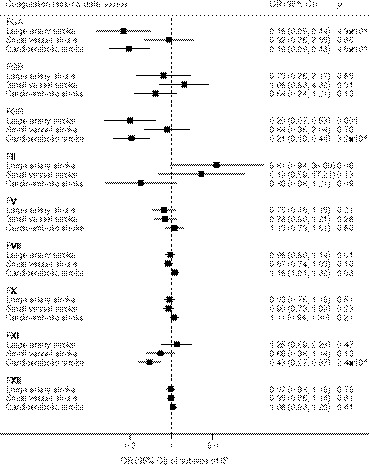
Genetically proxied inhibition of coagulation factors and subtypes of ischemic stroke (4373 large artery stroke, 5386 small vessel stroke and 7193 cardioembolic stroke cases from MEGASTROKE) in the main analysis. FGA indicates fibrinogen alpha chain; FGB, fibrinogen beta chain; FGG, fibrinogen gamma chain; and OR, odds ratio. Combined estimates were estimated using fixed‐effect meta‐analysis method.

Associations of coagulation factors with VTE and ischemic stroke and its subtypes are displayed in Figures 2, 3, 4 through 2, 3, 4. Other associations are displayed in Table 2. Genetically predicted prolonged aPTT was suggestively associated with heart failure, atrial fibrillation, and ischemic stroke, but not associated with other outcomes (Figure S1). There was no association between genetically predicted PT and cardiovascular outcomes (Figure S1).

**Table 2:** Genetically Proxied Inhibition of Coagulation Factors and Other Cardiovascular Outcomes

Data source	Cardiovascular Disease	FGA			FGB			FGG			FII			FV		
		OR	95% CI	*P* Value	OR	95% CI	*P* Value	OR	95% CI	*P* Value	OR	95% CI	*P* Value	OR	95% CI	*P* Value
UKBB	Coronary artery disease	1.09	0.76–1.57	0.65	0.97	0.68–1.39	0.86	0.93	0.66–1.32	0.69	1.07	0.57–2.00	0.84	0.99	0.84–1.17	0.94
CARDIoGRAMplusC4D	Coronary artery disease	0.86	0.60–1.25	0.43	1.08	0.75–1.55	0.68	0.84	0.59–1.19	0.33	0.91	0.49–1.69	0.77	0.88	0.76–1.02	0.10
UKBB	Heart failure	1.43	0.70–2.94	0.33	0.76	0.37–1.54	0.44	1.34	0.67–2.67	0.41	1.06	0.31–3.66	0.92	0.89	0.64–1.23	0.47
UKBB	Atrial fibrillation	0.98	0.62–1.55	0.93	1.22	0.77–1.93	0.39	0.91	0.59–1.42	0.69	0.62	0.28–1.36	0.23	0.79	0.64–0.98	0.03
UKBB	Aortic valve stenosis	0.53	0.16–1.80	0.31	1.53	0.46–5.07	0.49	0.48	0.15–1.54	0.22	0.72	0.09–5.96	0.76	0.96	0.55–1.69	0.89
UKBB	Abdominal aortic aneurysm	1.60	0.27–9.33	0.60	1.23	0.22–6.94	0.81	0.98	0.18–5.30	0.98	0.64	0.03–13.07	0.77	1.54	0.7–3.39	0.28
UKBB	Intracerebral hemorrhage	2.80	0.46–16.93	0.26	2.85	0.51–15.86	0.23	2.88	0.51–16.30	0.23	0.23	0.01–4.71	0.34	0.81	0.36–1.83	0.61
FinnGen	Intracerebral hemorrhage	1.94	0.40–9.4	0.41	0.26	0.05–1.39	0.12	1.50	0.33–6.76	0.60	0.37	0.03–4.81	0.45	1.16	0.62–2.15	0.65
UKBB	Subarachnoid hemorrhage	1.89	0.32–11.13	0.48	1.75	0.31–9.74	0.52	2.56	0.46–14.18	0.28	43.9	1.78–1080	0.02	2.13	0.99–4.58	0.05
FinnGen	Subarachnoid hemorrhage	0.43	0.08–2.39	0.33	4.96	0.81–30.25	0.08	0.32	0.06–1.63	0.17	0.32	0.02–5.15	0.42	1.41	0.72–2.78	0.32
UKBB	Peripheral arterial disease	0.78	0.29–2.11	0.63	1.12	0.42–3.00	0.82	0.74	0.29–1.93	0.54	1.68	0.30–9.45	0.56	0.95	0.61–1.48	0.82

Data source	Cardiovascular disease	FVII			FX			FXI			FXII		
		OR	95% CI	*P* Value	OR	95% CI	*P* Value	OR	95% CI	*P* Value	OR	95% CI	*P* Value
UKBB	Coronary artery disease	0.97	0.91–1.03	0.31	0.95	0.88–1.03	0.19	1.06	0.88–1.29	0.54	1.04	0.99–1.1	0.14
CARDIoGRAMplusC4D	Coronary artery disease	0.96	0.90–1.02	0.19	0.93	0.87–1.00	0.07	0.97	0.78–1.21	0.79	1.05	0.98–1.12	0.15
UKBB	Heart failure	1.09	0.97–1.24	0.16	1.06	0.91–1.23	0.45	1.03	0.70–1.51	0.89	1.00	0.90–1.11	1.00
UKBB	Atrial fibrillation	1.04	0.96–1.13	0.35	0.98	0.89–1.08	0.74	0.96	0.75–1.23	0.77	0.95	0.88–1.01	0.11
UKBB	Aortic valve stenosis	0.95	0.77–1.18	0.64	0.79	0.61–1.03	0.08	1.35	0.70–2.60	0.37	1.04	0.87–1.25	0.67
UKBB	Abdominal aortic aneurysm	0.91	0.67–1.25	0.57	1.01	0.70–1.46	0.94	1.07	0.42–2.74	0.89	0.98	0.75–1.27	0.87
UKBB	Intracerebral hemorrhage	0.78	0.57–1.08	0.13	0.92	0.64–1.35	0.68	1.5	0.58–3.88	0.41	1.10	0.85–1.43	0.46
FinnGen	Intracerebral hemorrhage	1.15	0.81–1.64	0.42	0.94	0.64–1.39	0.76	1.78	0.67–4.72	0.25	1.04	0.81–1.32	0.78
UKBB	Subarachnoid hemorrhage	1.15	0.86–1.56	0.34	1.19	0.83–1.70	0.35	2.01	0.78–5.18	0.15	0.82	0.63–1.07	0.14
FinnGen	Subarachnoid hemorrhage	1.45	0.99–2.13	0.05	1.20	0.79–1.83	0.40	0.47	0.16–1.36	0.16	0.87	0.67–1.14	0.32
UKBB	Peripheral arterial disease	0.89	0.75–1.06	0.20	0.91	0.74–1.13	0.40	1.23	0.72–2.10	0.45	1.02	0.88–1.18	0.83

CARDIoGRAMplusC4D indicates Coronary Artery Disease Genome‐wide Replication and Meta‐analysis; FGA, fibrinogen alpha chain; FGB, fibrinogen beta chain; OR odds ratio; and UKBB, UK Biobank.

Genetically proxied inhibition of the coagulation cascade via fibrinogen alpha, beta, and gamma chain and factors II and XI was significantly associated with reduced risk of VTE in the meta‐analysis of data from UK Biobank and FinnGen (*P*<0.001). The odds ratios scaled to a 1‐second increase in aPTT or PT were 0.04 (95% CI, 0.03–0.06) for fibrinogen alpha chain, 0.51 (95% CI, 0.34–0.77) for fibrinogen beta chain, 0.04 (95% CI, 0.03–0.06) for fibrinogen gamma chain, 0.16 (95% CI, 0.08–0.32) for factor II, and 0.15 (95% CI, 0.12–0.19) for factor XI. Genetically proxied inhibition of fibrinogen alpha and gamma chain and factor XI was also significantly associated with lower risk of ischemic stroke in the meta‐analysis of UK Biobank and MEGASTROKE data, and corresponding odds ratios were 0.36 (95% CI, 0.25–0.52), 0.37 (95% CI, 0.26–0.53), and 0.72 (95% CI, 0.58–0.88), respectively. With regard to subtypes of ischemic stroke, fibrinogen alpha and gamma and factor XI were associated with cardioembolic stroke (*P*<0.001). Fibrinogen alpha and gamma were further associated with large‐artery stroke (*P*<0.001). There were suggestive protective associations (*P*<0.05) of genetically proxied inhibition of fibrinogen beta and factors V, VII, and X with risk of ischemic stroke, and suggestive adverse effects of genetically proxied inhibition of factors II and XII on subarachnoid hemorrhage.

Phenotypes associated with variants used to proxy coagulation factor inhibition at the genome‐wide significance level are displayed in Table S5. As expected, most variants were associated with thrombosis‐related phenotypes, such as self‐reported deep vein thrombosis, pulmonary embolism, and phlebitis and thrombophlebitis, in genome‐wide association studies of European individuals. In addition, we found the effect allele of rs2059503 (inhibition of fibrinogen beta chain) to be associated with higher levels of aspartate transaminase, a marker of liver function. The effect allele of rs2070850 (inhibition of factor II) was associated with lower levels of high‐density lipoprotein cholesterol, bone mineral density and height, and lower liability to self‐reported hypertension. Rs2282686 (inhibition of factor II) was also associated with bone mineral density. The effect allele of rs9332653 (inhibition of factor V) was associated with lower blood protein levels, and the effect allele of 2 SNPs for factor XII inhibition was associated with increased height.

## Discussion

This MR study strengthens the evidence of causal associations of fibrinogen and factors II and XI with VTE. More importantly, we observed robust evidence that inhibition of fibrinogen and factor XI reduces the risk of ischemic stroke, particularly cardioembolic stroke. Additionally, we found suggestive evidence that inhibition of factors V, VII, and X might reduce the risk of ischemic stroke. These findings imply possible benefits of anticoagulant therapies targeting those coagulation factors in the prevention of ischemic stroke. We did not detect any significant associations of coagulation factors with other CVD outcomes, in contrast to evidence from clinical trials on factor Xa^22^ and thrombin inhibitors.^23^ A possible increased risk of hemorrhagic stroke could not be excluded for other anticoagulants and requires further study. Inhibition of factor VII, for example, may cause increased risk of severe intracranial hemorrhage.^24^

Overall, our findings were consistent with previous studies on the role of different coagulation factors in venous thrombosis.^25^, ^26^, ^27^ Identified protective effects of coagulant factor inhibition on ischemic stroke were also in agreement with meta‐analysis of clinical trials,^28^ and recent MR studies on factor XI.^29^, ^30^, ^31^ Notably, the present investigation went further to assess the effects of 9 coagulation factors on thrombosis and other CVD outcomes. Possible adverse effects of different anticoagulants should be considered further. As the CIs for the association with hemorrhagic stroke would still be compatible with clinically relevant risk increase, more evidence is needed to accurately estimate any potential impact of different coagulation factors on bleeding risk. This could be achieved by MR studies with greater statistical power (eg, larger number of hemorrhagic stroke cases) or other study designs.

Repurposing anticoagulant drugs for atherosclerotic CVD has been proposed in many large trials, albeit with inconclusive findings.^32^, ^33^ Specifically, effects of anticoagulants targeting factors II and X, such as dabigatran, rivaroxaban, apixaban, and edoxaban, on cardioembolic stroke have been identified. Our findings for factor X are compatible with the effects of previous clinical trials on the prevention of ischemic stroke but not with those on VTE,^34^ possibly attributable to pleiotropic effects of the genetic variants we employed. The F10 locus is located near the protein Z–dependent protease inhibitor gene, which shows a strong and independent association with ischemic stroke.^35^ The discrepancy may also be related by our application of genetic proxies for coagulation factors that were identified in Eastern Asian ancestry populations to CVD outcomes in European‐ancestry populations. Effects of anti–factor XI drugs on prevention of venous thrombosis have been studied in late‐stage trials.^36^ Given their potential protective effect on cardioembolic stroke highlighted in previous^29^, ^31^ MR studies as well as in our present work, whether such drugs can be used for cardioembolic stroke prevention may also warrant investigation in a clinical trial setting. In addition, and consistent with previous investigation, we also found evidence to support that factor VII may represent a therapeutic target for ischemic stroke.^37^ Phenome‐wide association studies on genetically proxied inhibition of coagulation factors represents a further approach to investigate the broad repurposing potential and adverse effect profile of anticoagulant drug classes.^38^ Current evidence on the role of fibrinogen is conflicting, with previous studies identifying relationships with both increased and decreased risk of venous thrombosis.^39^, ^40^ A recent MR study revealed that elevated fibrinogen gamma levels (based on 16 genetic instruments) and total fibrinogen levels (based on 75 genetic instruments) were associated with a decreased risk for thrombosis.^40^ Our findings, however, did not support these associations. The reasons underlying this discrepancy are unclear and may be related to our scaling of variant effects based on their relation to aPTT, while the previous study considered circulating fibrinogen levels.^40^ We note that coagulation factors may alter the risk of thrombosis by mechanisms that do not affect the aPTT, such as factor XIII activation and related fibrinolysis.^41^ In addition, we noticed that differences in the effects of 3 chains of fibrinogen on VTE. The fibrin clot is stabilized by activated factor XIII, which crosslinks gamma‐gamma and gamma‐alpha chains within the network.^42^ This crosslinking may explain a greater effect for variants at FGA and FGG as compared with FGB.

There are several strengths and limitations of the present study. The major merit is the MR design that reduced the bias introduced by unobserved confounding and reverse causation, and therefore strengthened the causal inference in the associations of 9 coagulation factors with CVD. In addition, associations were concordant in 2 independent data sources, which improved the robustness of our findings. We indirectly measured the effects of genetically proxied inhibition of coagulation factors through the association of genetic variants with either aPTT or PT, which limited the comparability of the magnitude of the associations of different coagulation factors with the outcomes. Future studies can adopt an alternative approach to identify variants by their relation to coagulation factor levels. As mentioned above, it may also be that the coagulation factors are exerting affects unrelated to aPTT or PT, which we are unable to measure. Another limitation is that association estimates using robust MR methods were not feasible because of a limited number of SNPs identified for each coagulation factor. Although we cannot rule out that our results might have been affected by potential pleiotropic effects, most incorporated variants were not strongly associated with established risk factors for VTE, stroke, or other CVDs. However, one of the SNPs for factor II and both SNPs for factor XII were associated with height, which is inversely associated with coronary artery disease, ischemic stroke, and peripheral artery disease and positively associated with VTE and atrial fibrillation.^43^ Thus, the association between genetically proxied inhibition of factor XII and coronary artery disease may be driven by pleiotropic effects related to height. Interestingly, some of the other associations may also be informative of potential adverse effects, such as related to bone mineral density and fracture risk, for example.^44^ Another limitation was that the negative findings might be attributed by lack of sufficient statistical power to detect an effect. However, it is impossible to estimate power in the present MR, as the unit of the effect of used SNPs for anticoagulation factors was not in SD units and the information on SD was unknown in the genome‐wide association studies we used. In general, there are concerns about performing post hoc power calculations (ie, after the analysis plan is fixed). The 95% CI for the estimates provides a useful indication of the plausible magnitude of causal effect based on the data available, and hence the extent to which findings are underpowered. Studies with large sample size are warranted to verify the null associations.

## Conclusions

The present MR study supports the efficacy of anticoagulants targeting fibrinogen, factor II, and factor XI in treating venous thromboembolism and revealed potential applications of inhibition of fibrinogen and factor XI for lowering risk of ischemic stroke, particularly cardioembolic stroke. We also noticed possible protective associations of genetically proxied inhibition of factors V, VII, and X with risk of ischemic stroke, which warrant further study. There was no significant evidence supporting effects on risk of other cardiovascular outcomes related to the use of anticoagulants. However, increased bleeding risk accompanied by these anticoagulants needs to be carefully assessed in further studies.

## Sources of Funding

Funding for this study came from the Karolinska Institutet’s Research Foundation Grants (Grant number 2020‐01842), the Swedish Research Council (Vetenskapsrådet; grant no. 2019‐00977), the Swedish Research Council for Health, Working Life and Welfare (Forte; grant no. 2018‐00123), the Swedish Heart‐Lung Foundation (Hjärt‐Lungfonden; grant no. 20190247), and the National Institute for Health Research (Cambridge Biomedical Research Centre at the Cambridge University Hospitals National Health Service Foundation Trust). Dipender Gill is supported by the British Heart Foundation Centre of Research Excellence (RE/18/4/34215) at Imperial College London and a National Institute for Health Research Clinical Lectureship at St. George's, University of London (CL‐2020‐16‐001). Amy M. Mason is supported by EC‐Innovative Medicines Initiative (BigData@Heart). Stephen Burgess is supported by a Sir Henry Dale Fellowship jointly funded by the Wellcome Trust and the Royal Society (grant no. 204623/Z/16/Z). Martin Dichgans is supported by the German Research Foundation (DFG) as part of the Munich Cluster for Systems Neurology (EXC 2145 SyNergy). This research was funded in part by the Wellcome Trust. For the purpose of open access, the author has applied a CC‐BY public copyright licence to any Author Accepted Manuscript version arising from this submission.

## Disclosures

Dr Gill is employed part‐time by Novo Nordisk, outside of the submitted work. The remaining authors have no disclosures to report.

## Supporting information


Tables S1‐S5
Figure S1
