# Supplementary material for: Genetically Proxied Inhibition of Coagulation Factors and Risk of Cardiovascular Disease: A Mendelian Randomization Study
Source: J Am Heart Assoc. 2021 Apr 9;10(8):e019644. doi: 10.1161/JAHA.120.019644 (PMC8174173; doi:10.1161/JAHA.120.019644)
Supplement: Supplementary file 1 — Tables S1‐S5 Figure S1 [file JAH3-10-e019644-s001.pdf]

Supplemental Material

Table S1. Detailed information on genetic instruments for coagulation factors.

Factor	Name	Indication	rsID	NEA	EA	EAF	Beta	SE	*p*
FI	Fibrinogen alpha	aPTT	rs6050	C	T	0.475	0.055	0.007	4.8E-14
FI	Fibrinogen beta	aPTT	rs2059503	A	T	0.128	0.065	0.011	3.1E-09
FI	Fibrinogen gamma	aPTT	rs2066861	T	C	0.491	0.059	0.007	7.3E-16
FII	Prothrombin	PT	rs2070850	T	C	0.551	0.040	0.007	1.5E-09
FV	Proaccelerin or labile factor	PT	rs9332678	T	A	0.596	0.079	0.006	1.3E-40
FV	Proaccelerin or labile factor	PT	rs6013	G	T	0.085	0.209	0.011	1.9E-87
FV	Proaccelerin or labile factor	PT	rs2239853	T	C	0.843	0.045	0.008	3.8E-08
FVII	Proconvertin or stable factor	PT	rs2774033	G	A	0.061	0.450	0.014	1.0E-200
FX	Stuart-Prower factor	PT	rs474810	T	C	0.017	0.394	0.024	1.6E-60
FXI	Plasma thromboplastin antecedent	aPTT	rs56810541	T	A	0.312	0.093	0.008	2.4E-28
FXII	Hageman factor	aPTT	rs4976649	A	G	0.247	0.338	0.010	1.0E-200
aPPT	aPPT	aPTT	rs6013	T	A	0.135	0.088	0.011	5.67E-16
aPPT	aPPT	aPTT	rs754549	G	T	0.085	0.172	0.013	4.33E-39
aPPT	aPPT	aPTT	rs5030081	C	T	0.399	0.059	0.008	3.92E-15
aPPT	aPPT	aPTT	rs1648717	A	G	0.722	0.121	0.008	3.97E-49
aPPT	aPPT	aPTT	rs149893292	A	G	0.505	0.059	0.007	4.31E-16
aPPT	aPPT	aPTT	rs12644950	G	A	0.290	0.072	0.008	1.22E-18
aPPT	aPPT	aPTT	rs56810541	T	A	0.688	0.093	0.008	2.40E-28
aPPT	aPPT	aPTT	rs4976649	G	A	0.175	0.068	0.010	6.20E-12
aPPT	aPPT	aPTT	rs55730132	G	C	0.690	0.225	0.008	4.18E-176
aPPT	aPPT	aPTT	rs7447593	A	G	0.753	0.338	0.010	1.00E-200
aPPT	aPPT	aPTT	rs28696310	A	G	0.337	0.052	0.008	8.66E-11
aPPT	aPPT	aPTT	rs687289	A	G	0.739	0.080	0.009	2.35E-18
aPPT	aPPT	aPTT	rs4962113	A	G	0.726	0.079	0.008	5.77E-21
aPPT	aPPT	aPTT	rs7870707	T	C	0.376	0.080	0.008	2.82E-26
aPPT	aPPT	aPTT	rs9411466	A	G	0.547	0.127	0.007	8.81E-68
aPPT	aPPT	aPTT	rs10793956	A	C	0.326	0.045	0.008	2.77E-08

aPPT	aPPT	aPTT	rs7895470	G	C	0.242	0.053	0.009	2.51E-09
aPPT	aPPT	aPTT	rs7962629	G	A	0.925	0.083	0.014	1.39E-09
aPPT	aPPT	aPTT	rs1801690	C	G	0.104	0.086	0.012	4.68E-13
PT	PT	PT	rs7521392	G	A	0.410	0.038	0.006	1.22E-10
PT	PT	PT	rs1208134	T	C	0.071	0.105	0.012	5.59E-20
PT	PT	PT	rs12022009	G	T	0.399	0.082	0.006	1.03E-41
PT	PT	PT	rs6013	G	T	0.085	0.209	0.011	1.85E-87
PT	PT	PT	rs2239853	T	C	0.843	0.045	0.008	3.78E-08
PT	PT	PT	rs1313566	G	A	0.436	0.037	0.006	3.33E-10
PT	PT	PT	rs2066861	T	C	0.509	0.054	0.006	2.04E-20
PT	PT	PT	rs2481942	A	G	0.060	0.090	0.014	2.65E-11
PT	PT	PT	rs927826	T	G	0.276	0.059	0.007	4.37E-19
PT	PT	PT	rs10761723	C	T	0.493	0.041	0.006	4.42E-12
PT	PT	PT	rs2070850	T	C	0.449	0.040	0.007	1.48E-09
PT	PT	PT	rs57799948	C	T	0.121	0.102	0.011	4.43E-22
PT	PT	PT	rs73576876	G	A	0.280	0.060	0.007	1.68E-19
PT	PT	PT	rs2181540	T	C	0.059	0.412	0.013	1.00E-200
PT	PT	PT	rs867186	G	A	0.959	0.150	0.015	5.64E-24

aPTT, indicates activated partial thromboplastin time; EA, effect allele; EAF, effect allele frequency; FGA, fibrinogen alpha-chain; FGB, fibrinogen beta-chain; FGG, fibrinogen gamma-chain; NEA, non-effect allele; PT, prothrombin time; SE, standard error. Beta estimate for each SNP was scaled to one-unit change in aPTT or PT.

Table S2. Definitions for cardiovascular disease outcomes in UK Biobank.

Outcome	Cases	Controls	ICD-9 diagnosis	ICD-10 diagnosis	OPCS procedure	Self-report†
Coronary artery disease	29 278	338 308	410, 411, 412, 414.0, 414.8, 414.9	I21, I22, I23, I24, I25.1, I25.2, I25.5, I25.6, I25.8, I25.9	K40, K41, K42, K43, K44, K45, K46, K49, K50.1, K50.2, K50.4, K75	20002, 20004, 6150
Heart failure	6712	360 874	402.01, 402.11, 402.91, 404.01, 404.11, 404.91, 404.03, 404.13, 404.93, 428	I11.0, I13.0, I13.2, I50		20002
Atrial fibrillation	16 945	350 641	427.3	I48		20002
Aortic valve stenosis	2244	365 342		I35.0, I35.2		20002
Abdominal aortic aneurysm	1094	366 492	441.3, 441.4	I71.3, I71.4	L19.4, L19.5	20002
Intracerebral hemorrhage	1064	366 522	431	I61		20002
Subarachnoid hemorrhage	1084	366 502	430	I60		20002
Ischemic stroke	4602	362 984	434, 436	I63, I64		20002
Venous thromboembolism	15 602	353 489	415.1, 451.1, 452, 453.0, 453.4, 453.9	I26, I80.1, I80.2, I81, I82.0	L90.2	20002, 6152
Peripheral arterial disease	3415	364 171	443.8, 443.9	I73.8, I73.9		20002

ICD, International Classification of Disease; OPCS, Office of Population Censuses and Surveys Classification of Surgical Operations and Procedures.
Follow-up for incident cases was until March 31, 2017 and date of death was recorded until February 14, 2018.
†Numbers refer to data fields used in UK Biobank: 6150/6152 = Health condition diagnosed by doctor (self-reported); 6177 = Medication for health condition (self-reported); 20002 = Non-cancer illness code (self-reported from interview with nurse); 20004 = Surgical operation code (self-reported from interview with nurse).

Table S3. Definitions for cardiovascular disease outcomes in FinnGen consortium.

Outcomes	Cases	Controls	Diagnostical information		
			ICD-10	ICD-9	ICD-8
Intracerebral hemorrhage	1224	163 533	I61	431	431
Subarachnoid hemorrhage	1019	163 508	I60	430	430
Venous thromboembolism	6913	169 986	O882/I80/O871/I26, excluding I800	415/451, excluding 4510	450|451|671|6739

ICD, International Classification of Disease. Information was obtained from the hospital discharge registry and cause of death registry.

Table S4. Definitions for cardiovascular disease outcomes in consortia
.

Outcomes	Consortium	Cases	Controls	Diagnostical information
Coronary artery disease	CARDIoGRAMplusC4D	60 801	123 504	Case status was defined by an inclusive coronary artery disease diagnosis (for example, myocardial infarction, acute coronary syndrome, chronic stable angina or coronary stenosis of >50%).
Any ischemic stroke	MEGASTROKE	60 341	NA	The stroke cases were defined as rapidly developing signs of focal (or global) disturbance of cerebral function, lasting more than 24 hours or leading to death with no apparent cause other than that of vascular origin. Any ischemic stroke was defined by all stroke cases except for intracerebral hemorrhage. Any ischemic stroke included large artery ischemic stroke, cardioembolic ischemic stroke, and small vessel ischemic stroke according to the Trial of Org 10,172 in Acute Stroke Treatment criteria, and also included ischemic stroke of undefined subtype.
Large artery stroke	MEGASTROKE	6688	146 392	
Small vessel stroke	MEGASTROKE	11 710	192 662	
Cardioembolic stroke	MEGASTROKE	9006	204 570	

NA, not available.

Table S5. Phenotypes associated with used SNPs at genome-wide significance†.

Factor	SNP	EA	Associated phenotype	Beta
FI	rs6050	T	Gamma fibrinogen levels	NA
			Plasma fibrin D dimer levels	NA
			Pulmonary embolism	-0.001
			Blood clot in the leg	-0.003
			Phlebitis and thrombophlebitis	-0.001
			Venous thrombosis	NA
FI	rs2059503	T	Aspartate transaminase	0.020
FI	rs2066861	C	Gamma fibrinogen levels	NA
			Self-reported DVT	-0.003
			Pulmonary embolism	-0.001
			Phlebitis and thrombophlebitis	-0.001
FII	rs2070850	C	High-density lipoprotein cholesterol	-0.053
			Height	-0.016
			Forced vital capacity	-0.020
			Heel bone mineral density	-0.029
			Self-reported hypertension	0.009
FV	rs9332678	A	None	
FV	rs6013	T	None	
FV	rs2239853	C	None	
FVII	rs2774033	A	None	
FX	rs474810	C	Factor VII clotting activity	NA
			Factor X antigen	NA
FXI	rs56810541	A	Self-reported DVT	-0.003
			Self-reported pulmonary embolism	-0.001
			Phlebitis and thrombophlebitis	-0.001
FXI	rs4253421	A	Self-reported pulmonary embolism	-0.003
			Self-reported DVT	-0.004
FXII	rs4976649	G	Height	0.013

†Pleiotropic phenotypes were obtained by a search in http://www.phenoscanner.medschl.cam.ac.uk/.
DVT, deep vein thrombosis; EA, effect allele; SNP, single-nucleotide polymorphism.

Figure S1. Genetically predicted aPTT and PT in relation to cardiovascular disease.

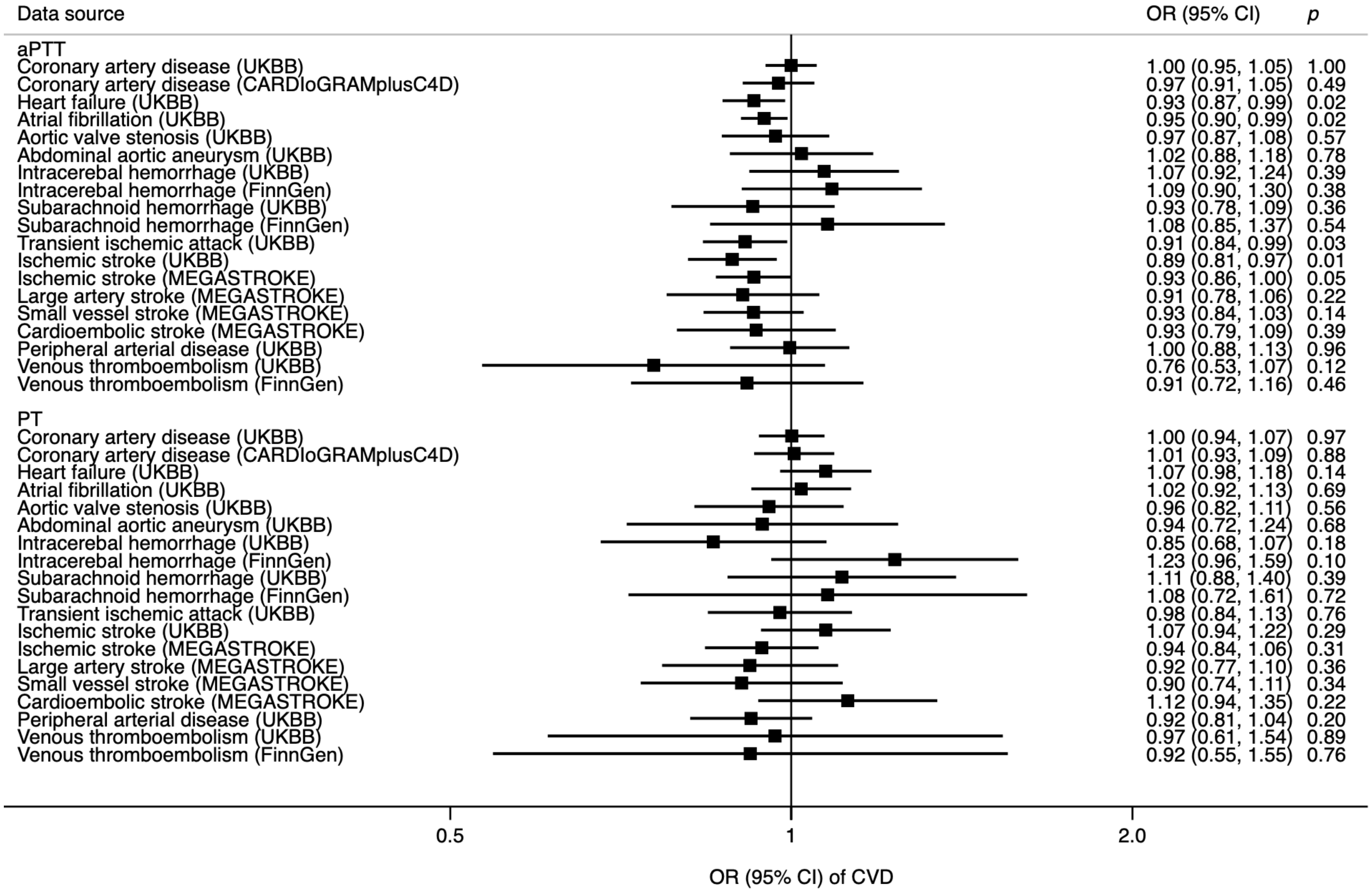

Activated partial thromboplastin time, aPTT; CI, confidence interval; CVD, cardiovascular disease; OR, odds ratio; prothrombin time, PT.
